# Determinants of vitamin a deficiency in children between 6 months and 2 years of age in Guinea-Bissau

**DOI:** 10.1186/1471-2458-13-172

**Published:** 2013-02-25

**Authors:** Niels Danneskiold-Samsøe, Ane Bærent Fisker, Mathias Jul Jørgensen, Henrik Ravn, Andreas Andersen, Ibraima Djogo Balde, Christian Leo-Hansen, Amabelia Rodrigues, Peter Aaby, Christine Stabell Benn

**Affiliations:** 1 Research Center for Vitamins and Vaccines (CVIVA), Bandim Health Project, Statens Serum Institut, Orestads Boulevard, Copenhagen S 2300, Denmark; 2 Bandim Health Project, Indepth Network, Bissau, Apartado 861 1004 Bissau Codex, Guinea-Bissau; 3 National Institute of Public Health (INASA), CP 1013, Bissau, Guinea-Bissau

**Keywords:** Vitamin A deficiency, Children, Guinea-Bissau, Risk factors, Retinol-binding protein

## Abstract

**Background:**

The World Health Organization (WHO) classifies Guinea-Bissau as having severe vitamin A deficiency (VAD). To date, no national survey has been conducted. We assessed vitamin A status among children in rural Guinea-Bissau to assess status and identify risk factors for VAD.

**Methods:**

In a vitamin A supplementation trial in rural Guinea-Bissau, children aged 6 months to 2 years who were missing one or more vaccines were enrolled, vaccinated and randomized to vitamin A or placebo. Provided consent, a dried blood spot (DBS) sample was obtained from a subgroup of participants prior to supplementation. Vitamin A status and current infection was assessed by an ELISA measuring retinol-binding protein (RBP) and C-reactive protein (CRP). VAD was defined as RBP concentrations equivalent to plasma retinol <0.7 μmol/L; infection was defined as CRP >5 ml/L. In Poisson regression models providing prevalence ratios (PR), we investigated putative risk factors for VAD including sex, age, child factors, maternal factors, season (rainy: June-November; dry: December-May), geography, and use of health services.

**Results:**

Based on DBS from 1102 children, the VAD prevalence was 65.7% (95% confidence interval 62.9-68.5), 11% higher than the WHO estimate of 54.7% (9.9-93.0). If children with infection were excluded, the prevalence was 60.2% (56.7-63.7). In the age group 9–11 months, there was no difference in prevalence of VAD among children who had received previous vaccines in a timely fashion and those who had not. Controlled for infection and other determinants of VAD, the prevalence of VAD was 1.64 (1.49-1.81) times higher in the rainy season compared to the dry, and varied up to 2-fold between ethnic groups and regions. Compared with having an inactivated vaccine as the most recent vaccine, having a live vaccine as the most recent vaccination was associated with lower prevalence of VAD (PR=0.84 (0.74-0.96)).

**Conclusions:**

The prevalence of VAD was high in rural Guinea-Bissau. VAD varied significantly with season, ethnicity, region, and vaccination status.

**Trial registration:**

Clinicaltrials.gov NCT00514891

## Background

Vitamin A deficiency (VAD) is a public health problem in many low-income countries. Worldwide, the prevalence of VAD is estimated to be 190 million in preschool-age children [1] causing 1–2 million deaths annually [2]. VAD is defined to be of public health importance if the national prevalence reaches 15% [3] using a serum or plasma retinol concentration < 0.7 μmol/L as cut-off for VAD [1,4]. Many countries lack data on vitamin A status. In Guinea-Bissau, no national vitamin A status survey has been conducted. Lacking biochemical vitamin A data, VAD estimates for preschool-age children are based on a regression model including the proxy variables: gross domestic product, under-5-mortality, and population growth rate. Using this method, Guinea-Bissau has been classified by the World Health Organization (WHO) as having a severe public health problem with an estimated 54.7% (9.9-93.0) of preschool-age children suffering from VAD [1]. Despite estimates of a high prevalence of VAD, little xerophthalmia has been observed in Guinea-Bissau [5]. In the present study we assessed the vitamin A status and investigated risk factors for VAD among 6-23-month-old children due to be vaccinated in rural Guinea-Bissau.

## Methods

### Study population

**Figure 1:**
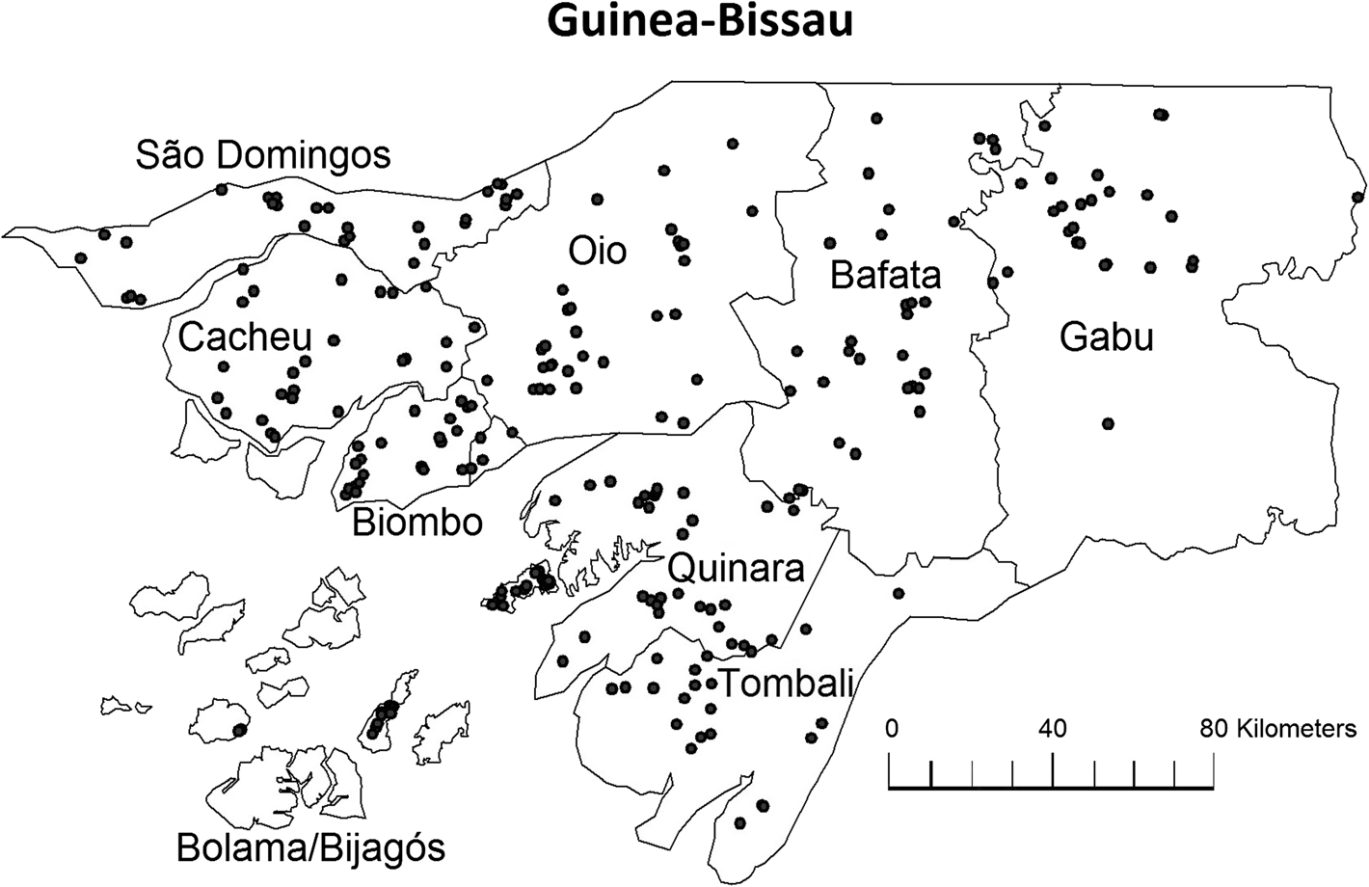
**Map of regions in Guinea-Bissau.** Villages included in the study marked as dots.

The Bandim Health Project (BHP) runs a Health and Demographic Surveillance System (HDSS) covering 182 randomly selected clusters of 100 women and their children below 5 years of age in the 9 rural health regions of Guinea-Bissau (Figure 1). The clusters are visited every 6 months by a team of field assistants. At each visit all women in the fertile age are interviewed to register new pregnancies and their children are followed until they reach 5 years of age. The following information is collected the first time a child is registered: sex of the child, date of birth, birth facility, maternal ethnicity, maternal education, and maternal age. At all visits, information is collected on breast feeding status, whether a bed net is used, and vaccination and vitamin A supplementation (VAS) history.

**Figure 2:**
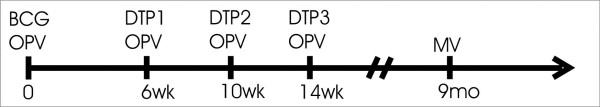
**Vaccine schedule in Guinea-Bissau.** Consisting of BCG vaccine, oral polio vaccine (OPV), diphtheria-tetanus-pertussis (DTP), and measles vaccine (MV). In September 2008 DTP was exchanged for diphtheria-tetanus-pertussis-Hepatitis B-H. *Influenzae* (Pentavalent) vaccine and yellow fever vaccine was introduced to be given together with MV. wk: weeks, mo: months.

The WHO recommended vaccination schedule in Guinea-Bissau is illustrated in Figure 2. The WHO policy of providing VAS at vaccination contacts after 6 months of age has not been implemented in Guinea-Bissau, but VAS is distributed during national campaigns. The present study was carried out within a randomized placebo-controlled trial testing the effect of providing VAS at the first vaccination contact after 6 months of age (trial registration: clinicaltrials.gov, number NCT00514891). The trial enrolled children from August 2007 to November 2010. Within the clusters all mothers were invited to participate in the trial if their child 1) was between 6 months and 2 years of age, 2) missed at least one routine vaccination, and 3) had not received VAS within the previous month (national VAS campaigns were conducted in July 2007, December 2007, July 2008, January 2009, July 2009, January 2010, and May 2010). No xerophthalmia was observed among the children.

At enrolment into the trial, after written informed consent was obtained by the present parent or guardian, the children were weighed and measured, and the mother was interviewed about whether she had given the child medicine and whether the child coughed, vomited or had diarrhea or fever on the day of enrolment.

We originally planned to collect blood samples from 600 children at enrolment, prior to randomization, to describe baseline vitamin A status among the children and study risk factors for VAD. The study was observational and no sample size calculations were made. In the beginning of the trial all eligible children in a village were included in the study. However, enrolment proceeded faster than expected and to describe the full seasonal and geographical variation we continued sampling five children from each village until samples from both the rainy (June-November) and the dry (December-May) season had been obtained in all regions. Hence, a total of 1254 samples were collected.

The study was in compliance with the Helsinki Declaration and was approved by the Ministry of Health in Guinea-Bissau. The Danish Central Ethical Committee provided its consultative approval.

### Handling and processing of blood samples

Dried blood spots (DBS) were prepared by letting finger prick blood drip onto filter paper (903 Protein Saver Card, Whatman International, UK). The papers were left to dry in a horizontal position protected from sunlight. After drying for a few hours, samples were bagged individually together with desiccant (Silica gel, Merck, NJ, USA) and left at ambient temperature for 1–14 days. Using this procedure, retinol-binding protein (RBP) levels remain stable for at least two weeks [6]. Upon arrival in the capital, samples were frozen at −20°C until analysis at the National Laboratory in Guinea-Bissau. Before analysis, samples were inspected for the presence of desiccant and dissociated plasma. Any plasma dissociation, seen as a yellow discoloration outside the DBS, or lack of desiccant was noted.

RBP and C-Reactive Protein (CRP) were measured using a previously developed sandwich enzyme-linked immunosorbent assay (ELISA) [7]. In brief, wells in two 96-well plates (Maxisorb C-shape, Nunc) were coated with polyclonal anti-human RBP antibody (A0040, Dako Denmark), and polyclonal anti-human CRP antibodies (Q0329, Dako, Denmark) respectively. DBS were eluted in buffer overnight and added to the wells after washing. After incubation and another round of washing, a second anti-human RBP (ICL, Dako, Denmark) and CRP antibody (Abcam, Dako, Denmark) conjugated to horseradish peroxidase was added. After a third round of incubation and washing, tetramethylbenzidine was added. After development of sufficient color, the reaction was stopped by adding sulphuric acid. Finally, the concentration was measured by spectrophotometry. All concentrations were measured in duplicates. If the coefficient of variation between two RBP measurements differed by > 20%, a second duplicate measurement was carried out. If the CRP value was ≥ 3 and ≤ 7 mg/L and the coefficient of variation between two CRP measurements differed by > 20%, a second duplicate measurement was carried out. If the CRP value was ≥ 7 or ≤ 3 mg/L and the coefficient of variation > 20%, duplicates differing more than 10% in optical density compared to the highest measured point on the standard curve were re-measured. A HPLC measured plasma sample was added as a quality control to each plate. If the quality control differed by more than 20% from the concentration measured by HPLC, the entire plate was re-measured.

### Determining vitamin a status

The blood concentration of retinol is under homeostatic control and remains stable over a large range of vitamin A storage concentrations [8]. Retinol is bound to RBP in the blood in a ≈1:1 complex but saturation tends to be lower with low retinol concentrations [9-11].

We used the WHO definition of subclinical VAD, which is a plasma retinol concentration of < 0.7 μmol/L. Previous estimates found by paired measurements of plasma retinol by HPLC and RBP by ELISA showed that a cut-off of 0.83 μmol/L for RBP was equivalent to a cut-off of 0.7 μmol/L for plasma retinol [9,12]. Infection lowers plasma retinol concentrations and acute phase proteins may be used to correct for overestimation of VAD due to infection [13]. We defined infection as a plasma concentration of CRP > 5 mg/L.

To estimate the cut-offs for VAD and infection using DBS, a total of 85 paired plasma and DBS samples were obtained and measured for RBP and CRP by ELISA. The paired measurements were used to form receiver operating characteristic curves. The areas under the curves were 0.75 and 0.90 for RBP and CRP respectively. The Youden index [14] was used to find the DBS cut-off value of 0.749 which corresponded to the highest combined sensitivity and specificity with regard to identifying individuals with VAD defined as plasma RBP < 0.83. The same was done for CRP, identifying the DBS cut-off which corresponded best, in terms of sensitivity and specificity, to a plasma CRP > 5 mg/L.

### Determining geography and distances

Global positioning system (GPS) coordinates were measured at hospitals, at vaccination facilities, and at the center of the village. Coordinates were loaded into ArcMap 9.3 (ESRI, Redlands, CA) and transformed into a Universal Transverse Mercator geographic coordinate system. An elevation of zero using the Shuttle Radar Topography Mission Digital Elevation Model (U.S. Geological Survey, Reston, VA) was used to define the sea. Euclidian distance in ArcMap was used to calculate the distances between villages and nearest vaccination facility, hospital, and the sea, respectively.

### Z-scores for anthropometry

Z-scores for weight-for-age, length-for-age, weight-for-length, and arm circumference-for-age were calculated using the WHO health growth standards [15] and the WHO Anthro version 3.1, June 2010 macro for Stata [16].

### Statistical analysis

First, we assessed the overall prevalence of VAD in all enrolled children. To correct for an overestimation due to infection, we subsequently conducted an analysis in which we omitted all children with infection. Next, in a sensitivity analysis, we assessed the prevalence of VAD using a 10% lower or 10% higher cut-off (0.749 ± 0.0749) in order to test the importance of the cut-off for VAD.

Since only children missing a vaccine according to the vaccination schedule were included in the study, the estimates of the prevalence of VAD may not entirely reflect vitamin A status in the population. The measles vaccine is supposed to be given at 9 months of age but children can be given this vaccine up to 12 months of age without this being considered as a late vaccination. We therefore defined a subgroup of children as being ”timely vaccinated” if they were 9–11 months of age, had received the third dose of DTP/Pentavalent vaccine before 7 months of age and were missing only MV (Figure 2). Next, we studied the prevalence of VAD within this subgroup and included it in our simple model for risk factors for VAD (see below).

We examined the risk factors for VAD in Poisson regression models, obtaining prevalence ratios (PR) for VAD [17]. For all continuous variables, we investigated whether there was evidence of a linear relationship between the variable and the prevalence of VAD by inspecting the prevalence of VAD in quintiles of the variable. If the association looked linear we tested for departure from linearity by inclusion of a quadratic term. Where evidence against linearity was not found, the variable was included as a continuous variable; otherwise the variable was included as categorical variables. A linear relationship with VAD could not be shown for the variables: length-for-age, distance to sea, distance to hospital, and distance to vaccination place.

For z-scores, the normal score was used as reference in the regression models. For distance variables, the lowest distance was used as reference. For the remaining variables the largest group was used as reference. Variables included in the analysis of risk factors for VAD are presented in Table 1. Variables were grouped under six headlines: obligatory variables (age, sex, and infection (CRP>5 mg/L)), child factors, season, geography, use of health services, and maternal factors (Table 1).

**Table 1:** Risk factors for vitamin A deficiency in rural Guinea-Bissau

**Group/Variable**	**n (%)**	**VAD Prevalence (%)**	**Simple model**^**a **^**PR for VAD (95% CI)**	**Simple model p**^**c **^**<**	**Large model**^**b **^**PR for VAD (95% CI)**	**Large model p**^**c **^**<**
VAD						
Yes	724 (66)	-	-	-	-	-
No	378 (34)	-	-		-	
***Obligatory***						
Sex						
Male	555 (50)	67	reference	0.39	reference	0.09
Female	547 (50)	64	0.96 (0.89-1.05)		0.93 (0.86-1.01)	
Age						
Continuous (months)	1102 (100)	-	0.99 (0.98-1.00)	0.05	1.00 (0.99-1.01)	0.83
CRP						
≤ 5 mg/L	756 (69)	60	reference	0.0001	reference	0.0001
> 5 mg/L	346 (31)	78	1.29 (1.19-1.40)		1.27 (1.17-1.37)	
***Child factors***						
Weight for age^d,e^						
Continuous	1094 (99)	-	0.98 (0.95-1.02)	0.42	1.05 (0.99-1.11)	0.13
Arm circumference for age^d,e^						
Continuous	1101 (100)	-	0.95 (0.91-0.99)	0.02	0.95 (0.89-1.01)	0.09
Length for age^d,e^						
< −2	267 (24)	62	1.01 (0.89-1.16)		-	-
≥ −2, < −1	325 (30)	67	0.93 (0.81-1.06)		-	-
≥ −1, < 0	345 (31)	67	0.99 (0.87-1.13)		-	-
≥ 0	161 (15)	50	reference	0.50	-	-
Weight for length^d,e^						
Continuous	1090 (99)	-	0.99 (0.95-1.02)	0.49	-	-
Twinning status						
Singletons	1066 (97)	65	reference	0.001	reference	0.01
Twins	36 (3)	86	1.31 (1.15-1.49)		1.30 (1.11-1.52)	
Breast feeding^d^						
Yes	1080 (98)	66	reference	0.51	-	-
Stopped	17 (2)	59	0.88 (0.61-1.27)		-	
Took medicine on inclusion day						
No	975 (88)	65	reference	0.05^f^	-	-
Yes	63 (6)	71	1.03 (0.88-1.22)		-	
No information	64 (6)	77	1.19 (1.03-1.37)		-	
Cough at the day of inclusion^d^						
No	816 (74)	64	reference	0.03	reference	0.03
Yes	283 (26)	72	1.11 (1.01-1.21)		1.10 (1.01-1.21)	
Fever at the day of inclusion^d^						
No	758 (69)	64	reference	0.42	-	-
Yes	341 (31)	69	1.04 (0.95-1.13)		-	
Diarrhea at the day of inclusion^d^						
No	920 (83)	65	reference	0.36	-	-
Yes	178 (16)	69	1.05 (0.94-1.17)		-	
***Maternal factors***						
Maternal ethnicity^d^						
Balanta	282 (26)	62	reference	0.0001	reference	0.001
Fula	215 (20)	81	1.31 (1.18-1.46)		1.21 (1.03-1.42)	
Mandinga	123 (11)	61	0.98 (0.83-1.16)		0.88 (0.75-1.03)	
Pepel	235 (21)	61	0.95 (0.83-1.08)		1.38 (1.05-1.81)	
Other^g^	234 (21)	62	1.00 (0.87-1.14)		0.95 (0.82-1.09)	
Maternal educational level (years of school)^d^						
None	797 (72)	66	reference	0.50	-	-
1-4	197 (18)	61	0.93 (0.83-1.05)		-	
> 4	101 (9)	67	1.01 (0.87-1.16)		-	
Maternal age at child inclusion date^d^						
≥ 14, ≤ 20	202 (18)	64	0.98 (0.87-1.10)		-	
> 20, ≤ 30	575 (52)	65	reference	0.74	-	-
> 30, ≤ 40	260 (24)	68	1.04 (0.94-1.15)		-	
> 40, ≤ 55	43 (4)	61	0.96 (0.75-1.22)		-	
***Season***						
Season						
Dry	649 (59)	53	reference	0.0001	reference	0.0001
Rainy	453 (49)	84	1.56 (1.44-1.70)		1.64 (1.49-1.81)	
***Geography***						
Region						
Bafata	121 (11)	55	1.02 (0.84-1.24)		1.41 (1.03-1.94)	
Biombo	287 (26)	56	reference	0.0001	reference	0.0001
Bijagós/Bolama	63 (6)	76	1.41 (1.19-1.66)		2.46 (1.79-3.39)	
Cacheu	53 (5)	43	0.81 (0.59-1.12)		1.47 (0.98-2.19)	
Gabu	148 (13)	82	1.47 (1.30-1.67)		2.10 (1.53-2.87)	
Oio	89 (8)	91	1.62 (1.44-1.83)		1.96 (1.49-2.58)	
Quinara	133 (12)	68	1.22 (1.04-1.42)		1.98 (1.48-2.66)	
Sao Domingos	91 (8)	65	1.15 (0.96-1.38)		1.87 (1.38-2.53)	
Tombali	117 (11)	63	1.13 (0.96-1.33)		2.07 (1.51-2.83)	
Village distance to vaccination facility (km)						
≥ 0, < 0.5	440 (40)	69	reference	0.10	reference	0.19
≥ 0.5, < 2	167 (15)	60	0.88 (0.77-1.01)		1.09 (0.94-1.26)	
≥ 2, < 5	200 (18)	61	0.88 (0.78-1.00)		1.07 (0.91-1.26)	
≥ 5	295 (27)	67	0.98 (0.89-1.09)		1.17 (1.01-1.36)	
Distance to sea (km)						
≥ 0, < 5	25 9 (24)	63	reference	0.001	-	-
≥ 5, < 15	290 (26)	60	0.96 (0.85-1.10)		-	
≥ 15, < 50	328 (30)	65	1.05 (0.93-1.19)		-	
≥ 50, < 149	225 (20)	77	1.22 (1.09-1.37)		-	
Distance to hospital (km)						
≥ 0, < 10	206 (19)	65	reference	0.03	reference	0.04
≥ 10, < 20	363 (33)	72	1.09 (0.97-1.22)		1.06 (0.93-1.20)	
≥ 20, < 30	319 (29)	61	0.94 (0.82-1.07)		0.93 (0.81-1.07)	
≥ 30, < 70	214 (19)	63	0.96 (0.83-1.11)		0.86 (0.72-1.02)	
***Use of health services***						
Most recent vaccination type^d,h^						
Inactivated	667 (61)	67	reference	0.93	reference	0.06
Live	141 (13)	64	0.96 (0.84-1.09)		0.84 (0.74-0.96)	
Mixed	156 (14)	66	1.00 (0.88-1.13)		1.01 (0.90-1.13)	
No vaccination	119 (11)	47	0.99 (0.86-1.14)		1.01 (0.88-1.17)	
VAS received within 6 months^d^						
Received	589 (53)	63	reference	0.12	-	-
Not received	500 (45)	68	1.07 (0.98-1.17)		-	
Birth facility						
Home	629 (57)	65	reference	0.01	reference	0.02
Health unit/center	80 (7)	58	0.89 (0.73-1.08)		0.98 (0.82-1.17)	
Hospital	126 (11)	77	1.20 (1.07-1.34)		1.16 (1.03-1.31)	
No information	267 (24)	64	1.00 (0.90-1.11)		1.13 (1.03-1.25)	
Child use of bed net^d^						
Year around	773 (70)	67	reference	0.03	reference	0.03
Rainy season	311 (28)	63	0.94 (0.85-1.04)		0.97 (0.87-1.09)	
None	8 (1)	88	1.38 (1.06-1.80)		1.48 (1.11-1.97)	

^a^Poisson regression adjusted for CRP > 5 mg/L, ^b^Poisson regression adjusted for CRP > 5 mg/L, sex, age, weight for age, arm circumference for age, twinning status, cough on inclusion day, maternal major ethnic group, season, region, village distance to vaccination facility, distance to hospital, most recent vaccination type, birth facility, and child use of bed net. ^c^Wald test for overall equality of categories within variable. ^d^Numbers do not add up to 1102 because records with missing information were discarded if they represent < 2%, ^e^z-score in standard deviations from 1. ^f^p=0.68 when children missing information was omitted, ^g^Grouped from minor ethnicities. ^h^Vaccination status determined by the most recent vaccination type being either live (BCG, OPV, MV, and/or yellow fever vaccine), inactivated (DTP or Pentavalent vaccine) or Mixed (both a live and an inactivated vaccine). OPV is supposed to be given with DTP vaccines, but the last vaccine type was not considered as mixed if OPV was given with a DTP or Pentavalent vaccine as done in previous studies.

All variables were tested one by one in a simple model, controlling only for infection. In a larger model we included the obligatory variables as well as all variables from the simple model, and used automated stepwise backwards selection to exclude variables to a descending value of p<0.20. Wald tests were used to test for overall equality of categories within variable. If a variable missed information for less than 2% of the participants, the missing category was excluded from both the simple and the large model. This was the case for the variables: weight for age, length for age, weight for length, breast feeding, cough at the day of inclusion, fever at the day of inclusion, diarrhea at the day of inclusion, maternal ethnicity, maternal educational level, maternal age, most recent vaccination type, VAS within 6 months, and child use of bed net (Additional file 1: Table S1).

To assess whether there was a temporal effect of previous VAS on vitamin A status we also tested the effect of including children who had received VAS within 2, 4, and 6 months prior to inclusion into the trial in the simple and the large model.

For all risk factors we evaluated whether the effect on VAD varied by sex and by infection status, respectively, by inclusion of an interaction term in both the simple and the large model. All interactions were evaluated by Wald statistics; a p-value below 0.05 was considered significant. Unless otherwise stated only the results of the large model are presented.

Finally we conducted a sensitivity analysis where we tested if the conclusions regarding risk factors for VAD changed if we a) excluded all children with infection, or b) used the 10% lower or higher cut-off for VAD.

All statistical analyses were conducted using Stata/SE 11.1.

## Results

A total of 1254 samples were collected between August 2007 and November 2009. Of the 1254 samples, eight samples were lost, two came from children who should not have been included, two samples did not have a corresponding inclusion questionnaire, 71 samples had been stored without desiccant, and 69 had dissociated plasma. Hence, 1102 samples were included in the study. Due to missing information on a variable (Additional file 1: Table S1) the final number of children included in the large model was 1050.

### Overall prevalence of VAD and infection

The overall prevalence of VAD was 65.7% (95% CI=62.9-68.5). The prevalence of infection was 31.4% (28.7-34.4). If we excluded all children with infection, the prevalence of VAD was 60.2% (56.7-63.7). Using a 10% lower cut-off it was 57.5% (54.6-60.5) and using a 10% higher cut-off it was 73.5% (70.9-76.1).

### Risk factors for VAD

Sex of the child had no significant effect on the prevalence of VAD but girls tended to have slightly lower prevalence of VAD, the PR for VAD being 0.93 (0.86-1.01) (Table 1). The risk of VAD tended to decrease with increasing age in the simple model. However, the marginal effect disappeared after adjustment (Table 1). As expected, having an infection was associated with an increased risk of VAD, the PR being 1.27 (1.17-1.37).

#### Child factors

Arm circumference was associated with VAD in the simple model, but not in the large model after adjustment (Table 1). None of the other anthropometric variables were associated with VAD. Twins had a higher risk of VAD (the PR being 1.30 (1.11-1.52)), while breastfeeding had no effect on VAD. Cough on the inclusion day was positively associated with VAD (PR=1.10 (1.01-1.21)), whereas fever and diarrhea and use of medicine on the date of enrolment were not (Table 1).

#### Maternal factors

Fula and Pepel ethnicity were associated with an increased prevalence of VAD compared with Balanta ethnicity (PR=1.21 (1.03-1.42) and 1.38 (1.05-1.81), respectively) (Table 1). Maternal educational level and age were not associated with VAD.

#### Season

**Figure 3:**
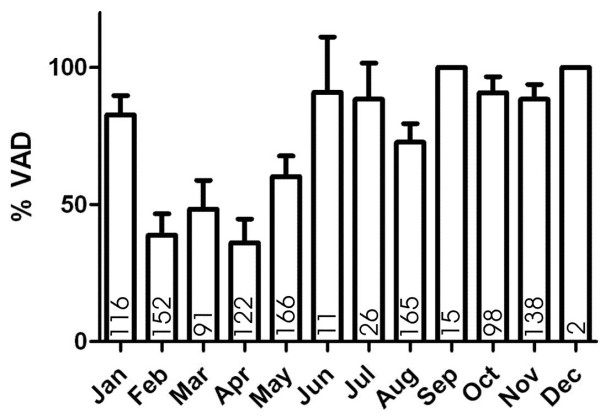
**Prevalence of VAD by month in Guinea-Bissau.** Number of samples tested indicated in the lower part of each bar.

Rainy season (June to November) was associated with a high risk of VAD, the PR being 1.64 (1.49-1.81) compared with the dry season (December-May). The lowest risk of VAD was found at the end of the dry season, from February to May, where the prevalence of VAD was 46.5% (42.3-50.8) (Figure 3).

#### Geography

There were considerable differences in the prevalence of VAD between the nine health regions (Figure 1 and Table 1). The regions of Bijagós/Bolama, Gabu, and Tombali had a more than 2-fold higher prevalence of VAD compared to the region of Biombo which is closest to the capital (the PRs being 2.46 (1.79-3.39), 2.10 (1.53-2.87), 2.07 (1.51-2.83) respectively). A distance equal to or above 5 km to a vaccination facility did not increase the risk of VAD in the simple model but were associated with a higher risk in the large model (PR=1.17 (1.01-1.36)). Although distance to the sea was associated with a higher prevalence of VAD in the simple model (Table 1), no linear relationship with VAD was found, and due to confounding by region the variable was not included in the large model. Distance to hospital was associated with VAD, but in a non-linear manner (Table 1).

#### Use of health services

Having received a live vaccine as the most recent vaccine rather than an inactivated vaccine was associated with a lower risk of VAD (PR= 0.84 (0.74-0.96) (Table 1)). We did not find an effect on the risk of VAD of having received VAS within the previous 6 months (Table 1). VAS within 2 or 4 was not associated with significantly lower risk of VAD either (Additional file 1: Table S2). Hospital delivery was associated with an increased risk of VAD (PR=1.16 (1.03-1.31)) compared to home delivery. The eight children who did not use bed net at any time of the year had a higher risk of VAD than children who used bed nets all year (PR=1.48 (1.11-1.97)).

A total of 380 children were 9–11 months of age. Of these, 124 children were timely vaccinated. The prevalence of VAD was 61% in the group of timely vaccinated children and 69% in those who had not been timely vaccinated. The risk of VAD was not significantly different between the two groups (PR=0.92 (0.76-1.10) in the timely vaccinated versus the non-timely vaccinated (Additional file 1: Table S3).

#### Interactions between risk factors and sex and infection

We found no interactions between sex and any of the risk factors retained in the large model. There were significant interactions between infection and weight for age (p<0.0001), ethnicity (p<0.001), season (p<0.01), and region (p<0.001), respectively; in children with infection the associations between these risk factors and VAD were less pronounced.

#### Sensitivity analysis

If children with infection were excluded, retaining 756 children in the analysis, cough on inclusion became insignificant in both models, arm-circumference-for-age was no longer significantly associated with VAD in the simple model, and most recent vaccination type became statistically significant in the large model (Additional file 1: Table S4).

Using the low cut-off for VAD thereby retaining only the most deficient children by setting the cut-off for VAD lower than 0.83, identified the same main risk factors for VAD in the large model with a few exceptions. Twinning status was no longer a risk factor and birth facility, and distance to hospital was no longer risk factors in any of the models (Additional file 1: Table S5).

Using a high cut-off for VAD also identified the same main risk factors in the large model except for birth facility that was no longer significant in the large model, and the use of bed net that was no longer significant in the both models (Additional file 1: Table S6).

## Discussion

### Main findings

Based on the collected blood samples young children in rural Guinea-Bissau may have a major VAD problem. The overall prevalence of VAD was 66%, 11% higher than the WHO estimate. Infection, twinning, cough on inclusion day, Fula and Pepel ethnicity, rainy season, living in the regions of Bijagós/Bolama, Gabu, and Tombali, and hospital delivery were associated with an increased risk of VAD. Receiving a live vaccine as the most recent vaccine was associated with a lower prevalence of VAD compared with having received an inactivated vaccine as the most recent vaccine. These factors remained significant or became even more pronounced when children with infection were excluded (Additional file 1: Table S5).

### Strengths and weaknesses

No national vitamin A status study has been conducted in Guinea-Bissau and the present study is the first to describe the determinants of VAD. Using a DBS assay approach allowed for the collection of samples from many areas within the country, which would not have been possible if we had used plasma or serum samples due to lack of electricity.

A limitation that may possibly contribute to an overestimation of VAD in the study is the incomplete correction for infection by not including α1-acid-glycoprotein, and/or another slow acute phase protein in the models [13].

In the present study we took advantage of the infrastructure of a trial and only children eligible for the trial were bled. Thus, children were between 6–23 months of age and missed at least one routine vaccination, and no children were included in the first month after VAS provided in a campaign. Children with incomplete vaccination history have been shown to have a higher risk of VAD in other settings [18]. However, in our study population in rural Guinea-Bissau it is common to be missing one or more vaccines; in a recent study from the Bandim Health Project we found that half of the children at one year of age in Guinea-Bissau are missing at least one vaccine [19]. Furthermore, 67% of children in the age group from 9–11 months of age had not yet received a measles vaccine at the time of the home visit, and we did not find any statistical difference in the risk of VAD between the children who had received all the prior vaccines timely and children who had not. Hence, though we cannot exclude a slight overestimation of VAD due to the focus on children missing one or more vaccines, we believe that the study reflects the situation in rural Guinea-Bissau.

Since study children did not receive VAS within the preceding month, a short-lived increase in serum RBP after supplementation in a campaign is not likely to have affected the results [20]. On the other hand, we might have overestimated the prevalence of VAD by not including this group. However, we were not able to document any effect of receiving VAS from 1 to 2 months before inclusion.

All geographic areas were represented in the sample. However, the included populations did not completely correspond to the respective population sizes in the various regions. Due to the inter-regional variation in risk of VAD this could have affected the overall estimate of VAD prevalence. However, calculating a weighted average the combined prevalence from all regions was 67%. The four months with the lowest prevalence of VAD corresponded to 48.2% of the study population, which will tend to underestimate the prevalence of VAD.

In conclusion, we may not have obtained a precise estimate of the true prevalence of VAD in the children in Guinea-Bissau, but neither setting the cut-off for VAD lower, nor exclusion of infected children or estimating the prevalence of VAS in timely vaccinated children resulted in a prevalence below 57.5% as estimated by the WHO. The analysis of determinants of VAD was also robust in the sensitivity analyses.

### Consistency with other studies

Several studies of vitamin A status in children have investigated determinants for VAD. Since the Bandim Health Project have previously observed sex differences in the effect of vitamin A supplementation [5,21-23] sex was included as an obligatory variable in the analysis. However, most studies have found no sex differences of VAD [9,24,25] and our study is in line with this, though with a tendency towards lower VAD in girls.

In countries where VAD is a public health problem, the WHO recommends VAS every 4–6 months [26]. Several trials have evaluated the temporal effect of supplementation on VAD, but none including children within the study age group. We found only two studies investigating the effect of VAS on vitamin A status in the present age group. An observational study from Ethiopia reported increased VAD in children who had missed one or more doses of VAS within the last year [18]. The other observational study collected paired blood samples before and after supplementation and found a significant increase in plasma retinol 4 months after VAS [27]. We could not document any effect of missing VAS within the preceding 2, 4 or 6 months. Possible explanations could be geographical or methodological differences between the studies. The study from Ethiopia also reported an effect on VAD of missing a vaccine and coming from a Muslim household. We did not find a consistent effect of coming from a Muslim household. The Fulas are Muslims and had a high prevalence of VAD, but so are the Mandingas, who did not have a high prevalence of VAD.

Seasonal differences in vitamin A status have been documented in West Africa before, both in children [28] and in lactating and pregnant mothers [29,30]. Our results also show strong seasonal differences, and these were particularly pronounced in children without infection.

We have previously shown that the inactivated DTP vaccine given after neonatal VAS was associated with reduced RBP levels in 4 months old girls [9]. In the present study the prevalence of VAD was higher if the most recent vaccine was DTP compared with a live vaccine (MV, BCG, OPV), but the effect did not differ by sex in these older children.

### Interpretation

The important dietary sources of pre-formed vitamin A are animal products such as egg and liver. In addition, β-carotene, which can be found in orange-yellow vegetables and fruits such as mango as well as in red palm oil [31-33] can be converted to retinoids [34]. All of the above foods can be found in Guinea-Bissau. We found strong seasonal variation in the prevalence of VAD and a possible explanation could be food availability. Mango supply might partly explain seasonal variation in vitamin A status, either directly or through breastfeeding [29,35] although the mango season starts a month later than the months with a low prevalence of VAD. The decreased prevalence of VAD in the dry season could also be explained by the harvest in December-January. Underestimation of infection may also partly explain why season is a strong predictor but this seems unlikely since no significant difference in the prevalence of a high CRP between seasons was found (30% (27–34) and 33% (29–37) in the dry and rainy season, respectively). It seems likely that vitamin A status follows the intake of vitamin A rich foods and to a lesser degree VAS since season was a strong predictor for VAD, but VAS within the preceding months was not. If the WHO guidelines were to be followed, special attention should be given to improving vitamin A status in risk situations, e.g. in the rainy season. However, several studies from Guinea-Bissau have shown that supplementing with vitamin A in the rainy season may have a negative impact on overall mortality [20,36]. This emphasizes that VAS may have other effects than merely prevention of VAD.

The reason for the strong regional differences is not clear. Distance to the sea was confounded by region and might explain the high risk of VAD in the region of Gabu. We have no explanation why hospital deliveries were associated with a higher risk of VAD; this might be a spurious finding.

The increased risk of VAD amongst twins could possibly be explained by a lower nutritional status since twins are more often born low-birth-weight and run a higher risk of being stunted [37]. However, due to the low number of twins (n=36) the result should be interpreted with caution.

The lower prevalence of VAD among children who had a live vaccine as their most recent vaccine supports previous observations that live vaccines are associated with beneficial effect on child health [38,39].

## Conclusion

We found that around two-thirds of children between 6 months and 2 years of age have RBP levels indicating VAD. Though we only included children who were not fully vaccinated, this accounts for a large proportion of the children in rural Guinea-Bissau, and there was no indication that children vaccinated timely had significantly lower risk of VAD. Though we may not have been able to control fully for the effect of infections, the results still point to VAD as a major public health problem in rural Guinea-Bissau. In our analysis of determinants we found that major risk factors were season, region, ethnicity, and vaccination status. The prevalence of VAS varied up to 2-fold between different groups, hence it is possible to identify subgroups of children at particular risk. In light of the missing effects of VAS on vitamin A status it may be better to opt for other methods to improve vitamin A status, i.e. through fortification, promoting vitamin A rich foods or more frequent low-dose supplementation.

## Abbreviations

DBS: Dried Blood Spot; DTP: Diphtheria-tetanus-pertussis; ELISA: Enzyme-Linked ImmunoSorbent Assay; GPS: Global Positioning System; HDSS: Health and Demographic Surveillance System; MV: Measles vaccine; Pentavalent: Diphtheria-tetanus-pertussis-hepatitis B-*H. influenzae* type B; PR: Prevalence Ratio; RBP: Retinol Binding Protein; VAD: Vitamin A deficiency; VAS: Vitamin A supplementation; WHO: World Health Organization.

## Competing interests

No competing interests exist. This manuscript has not been published before or submitted elsewhere for publication.

## Authors’ contributions

NDS: laboratory work, data analysis and the draft of the manuscript; ABF: conception of the research idea, study design, data collection and analysis, interpreted the data and review of manuscript; MJJ: conception of the research idea, study design, data collection, part of laboratory work; HR, AA: data analysis and reviewed manuscript; IDB: data collection and analysis; CLH: laboratory work and review of manuscript, AR: conception of the research idea, study design, data collection; PA, CB: conception of the research idea, study design, interpreted the data and review of manuscript. All authors have read and approved the final version of the manuscript.

## Pre-publication history

The pre-publication history for this paper can be accessed here:

http://www.biomedcentral.com/1471-2458/13/172/prepub

## Supplementary Material

Additional file 1: Table S1 Overview of missing data in included variables. Supplementary **Table S2** Effect of receiving VAS within 2 or 4 months prior to inclusion if included in the simple and large model respectively. Supplementary **Table S3** Risk of VAD in timely vaccinated children if included in the simple and large model compared to other children within the same age group. Supplementary **Table S4** Risk factors for vitamin A deficiency in rural Guinea-Bissau for children without infection. Supplementary **Table S5** Risk factors for vitamin A deficiency in rural Guinea-Bissau retaining only the most deficient children by setting the cut-off for VAD 10% lower than 0.83. Supplementary table S6 Risk factors for vitamin A deficiency in rural Guinea-Bissau retaining both the most and marginally deficient children by setting the cut-off for VAD 10% higher than 0.83. Click here for file
