# Supplementary material for: Determinants of vitamin a deficiency in children between 6 months and 2 years of age in Guinea-Bissau
Source: BMC Public Health. 2013 Feb 25;13:172. doi: 10.1186/1471-2458-13-172 (PMC3599523; doi:10.1186/1471-2458-13-172)
Supplement: Additional file 1: Table S1 — Overview of missing data in included variables. Supplementary Table S2 Effect of receiving VAS within 2 or 4 months prior to inclusion if included in the simple and large model respectively. Supplementary Table S3 Risk of VAD in timely vaccinated children if included in the simple and large model compared to other children within the same age group. Supplementary Table S4 Risk factors for vitamin A deficiency in rural Guinea-Bissau for children without infection. Supplementary Table S5 Risk factors for vitamin A deficiency in rural Guinea-Bissau retaining only the most deficient children by setting the cut-off for VAD 10% lower than 0.83. Supplementary table S6 Risk factors for vitamin A deficiency in rural Guinea-Bissau retaining both the most and marginally deficient children by setting the cut-off for VAD 10% higher than 0.83. [file 1471-2458-13-172-S1.docx]

**Supplementary tables**

**Vitamin A status between 6 months and 2 years of age in Guinea-Bissau**

**Supplementary table S1.** Overview of missing data in included variables

| **Variable** | **Missing data** |
| --- | --- |
| Variables not included in the large model |  |
| Length for age | 4 |
| Weight for length | 12 |
| Breast feeding | 5 |
| Fever at the day of inclusion | 3 |
| Diarrhea at the day of inclusion | 4 |
| Maternal educational level | 7 |
| Maternal age | 22 |
| VAS received within 6 months | 13 |
| Variable included in the large model |  |
| Weight for age^a^ | 8 |
| Arm circumference for age | 1 |
| Cough at the day of inclusion | 3 |
| Maternal ethnicity^b^ | 13 |
| Most recent vaccination type^a^ | 19 |
| Child use of bed net^b^ | 10 |
| Final inclusions into larger model | 1050 |

^a^one inclusion with data missing in both variables: weight for age and most recent vaccination type, ^b^one inclusion with data missing in both variables: maternal ethnicity and child use of bed net.

| **Group/Variable** | **n (%)** | **VAD Prevalence (%)** | **Simple model^a^ PR for VAD (95%CI)** | **Simple model p^c^ <** | **Large model^b^ PR for VAD (95%CI)** | **Large model p^c^ <** |
| --- | --- | --- | --- | --- | --- | --- |
| VAS^d^ |  |  |  |  |  |  |
| Received ≤ 2 months | 379 (34) | 64 | 0.98 (0.89-1.07) |  | 0.95 (0.87-1.04) |  |
| > 2 months or none | 710 (64) | 66 | reference | 0.60 | reference | 0.26 |
|  |  |  |  |  |  |  |
| Received ≤ 4 months | 493 (45) | 63 | 0.94 (0.86-1.02) |  | 0.97 (0.89-1.06) |  |
| > 4 months or none | 596 (54) | 68 | reference | 0.15 | reference | 0.53 |

**Supplementary table S2.** Effect of receiving VAS within 2 or 4 months prior to inclusion if included in the simple and large model respectively

^a^Poisson regression adjusted for CRP > 5 mg/L, ^b^Poisson regression adjusted for CRP > 5 mg/L, sex, age, weight for age, arm circumference for age, twinning status, cough on inclusion day, maternal major ethnic group, season, region, village distance to vaccination facility, distance to hospital, most recent vaccination type, birth facility, and child use of bed net. ^c^Wald test for overall equality of categories within variable. ^d^Numbers do not add up to 1102 because of 13 children with missing VAS data.

| **Group/Variable** | **n (%)** | **VAD Prevalence (%)** | **Simple model^a^ PR for VAD (95%CI)** | **Simple model p^c^ _<_** | **Large model^b^ PR for VAD (95%CI)** | **Large model p^c^ <** |
| --- | --- | --- | --- | --- | --- | --- |
| Timely vaccinated |  |  |  |  |  |  |
| Yes | 124 (11) | 61 | 0.89 (0.76-1.05) |  | 0.92 (0.76-1.10) |  |
| No | 256 (23) | 70 | reference | 0.16 | reference | 0.34 |

**Supplementary table S3.** Risk of VAD in timely vaccinated children if included in the simple and large model compared to other children within the same age group

^a^Poisson regression adjusted for CRP > 5 mg/L, ^b^Poisson regression adjusted for CRP > 5 mg/L, sex, age, weight for age, arm circumference for age, twinning status, cough on inclusion day, maternal major ethnic group, season, region, village distance to vaccination facility, distance to hospital, most recent vaccination type, birth facility, and child use of bed net. ^c^Wald test for overall equality of categories within variable.

**Supplementary table S4.** Risk factors for vitamin A deficiency in rural Guinea-Bissau for children without infection

| **Group/Variable** | **n (%)** | **VAD Prevalence (%)** | **Simple model^a^ PR for VAD (95%CI)** | **Simple model p^c^ <** | **Large model^b^ PR for VAD (95%CI)** | **Large model p^c^ <** |
| --- | --- | --- | --- | --- | --- | --- |
| VAD |  |  |  |  |  |  |
| Yes | 455 (60) | - | - | - | - | - |
| No | 301 (40) | - | - |  | - |  |
| ***Obligatory*** |  |  |  |  |  |  |
| Sex |  |  |  |  |  |  |
| Male | 377 (50) | 61 | reference | 0.65 | reference | 0.36 |
| Female | 379 (50) | 59 | 0.97 (0.87-1.09) |  | 0.95 (0.85-1.06) |  |
| Age |  |  |  |  |  |  |
| Continuous (months) | 756 (100) | - | 0.99 (0.97-1.00) | 0.09 | 1.00 (0.98-1.01) | 0.50 |
| CRP |  |  |  |  |  |  |
| ≤ 5 mg/L | 756 (100) | 60 | - | - | - | - |
| > 5 mg/L | 0 (0) | - | - |  | - |  |
| ***Child factors*** |  |  |  |  |  |  |
| Weight for age^d,e^ |  |  |  |  |  |  |
| Continuous | 751 (99) | - | 1.01 (0.96-1.06) | 0.77 | 1.07 (0.98-1.16) | 0.13 |
| Arm circumference for age^d,e^ |  |  |  |  |  |  |
| Continuous | 755 (100) | - | 0.96 (0.91-1.02) | 0.18 | 0.97 (0.89-1.06) | 0.48 |
| Length for age^d,e^ |  |  |  |  |  |  |
| < -2 | 182 (24) | 61 | 0.98 (0.81-1.18) |  | - | - |
| ≥ -2, < -1 | 234 (31) | 56 | 0.90 (0.75-1.08) |  | - | - |
| ≥ -1, < 0 | 225 (30) | 63 | 1.02 (0.85-1.21) |  | - | - |
| ≥ 0 | 111 (15) | 62 | reference | 0.45 | - | - |
| Weight for length^d,e^ |  |  |  |  |  |  |
| Continuous | 747 (99) | - | 1.00 (0.95-1.05) | 0.99 | - | - |
| Twinning status |  |  |  |  |  |  |
| Singletons | 733 (97) | 60 | reference | 0.02 | reference | 0.05 |
| Twins | 23 (3) | 78 | 1.31 (1.05-1.64) |  | 1.26 (1.01-1.57) |  |
| Breast feeding^d^ |  |  |  |  |  |  |
| Yes | 740 (98) | 61 | reference | 0.20 | - | - |
| Stopped | 11 (2) | 36 | 0.60 (0.27-1.31) |  | - |  |
| Took medicine on inclusion day |  |  |  |  |  |  |
| No | 682 (90) | 59 | reference | 0.16^f^ | - | - |
| Yes | 28 (4) | 61 | 1.02 (0.75-1.39) |  | - |  |
| No information | 46 (6) | 72 | 1.21 (1.00-1.46) |  | - |  |
| **Supplementary table S4 continued** | | | | | | |
| Cough at the day of inclusion^d^ |  |  |  |  |  |  |
| No | 579 (77) | 59 | reference | 0.25 | reference | 0.55 |
| Yes | 174 (23) | 64 | 1.08 (0.95-1.23) |  | 1.04 (0.92-1.18) |  |
| Fever at the day of inclusion^d^ |  |  |  |  |  |  |
| No | 555 (73) | 60 | reference | 0.75 | - | - |
| Yes | 198 (26) | 61 | 1.02 (0.90-1.16) |  | - |  |
| Diarrhea at the day of inclusion^d^ |  |  |  |  |  |  |
| No | 642 (85) | 60 | reference | 0.40 | - | - |
| Yes | 110 (15) | 64 | 1.07 (0.92-1.25) |  | - |  |
| ***Maternal factors*** |  |  |  |  |  |  |
| Maternal ethnicity^d^ |  |  |  |  |  |  |
| Balanta | 196 (26) | 55 | reference | 0.0001 | reference | 0.01 |
| Fula | 158 (21) | 77 | 1.40 (1.20-1.63) |  | 1.41 (1.13-1.76) |  |
| Mandinga | 86 (11) | 58 | 1.06 (0.85-1.31) |  | 0.99 (0.80-1.21) |  |
| Pepel | 141 (19) | 50 | 0.91 (0.74-1.12) |  | 1.54 (1.09-2.17) |  |
| Other^g^ | 166 (22) | 58 | 1.05 (0.88-1.26) |  | 1.03 (0.85-1.24) |  |
| Maternal educational level (years of school)^d^ |  |  |  |  |  |  |
| None | 541 (72) | 61 | reference | 0.56 | - | - |
| 1-4 | 146 (19) | 56 | 0.92 (0.79-1.08) |  | - |  |
| > 4 | 66 (9) | 62 | 1.02 (0.83-1.24) |  | - |  |
| Maternal age at child inclusion date^d^ |  |  |  |  |  |  |
| ≥ 14, ≤ 20 | 131 (17) | 57 | 0.96 (0.81-1.13) |  | - |  |
| > 20, ≤ 30 | 398 (53) | 60 | reference | 0.76 | - | - |
| > 30, ≤ 40 | 177 (23) | 63 | 1.05 (0.91-1.21) |  | - |  |
| > 40, ≤ 55 | 34 (5) | 56 | 0.93 (0.69-1.27) |  | - |  |
| ***Season*** |  |  |  |  |  |  |
| Season |  |  |  |  |  |  |
| Dry | 453 (60) | 46 | reference | 0.0001 | reference | 0.0001 |
| Rainy | 303 (40) | 81 | 1.76 (1.57-1.97) |  | 1.85 (1.62-2.10) |  |
| ***Geography*** |  |  |  |  |  |  |
| Region |  |  |  |  |  |  |
| Bafata | 101 (13) | 52 | 1.15 (0.90-1.47) |  | 1.56 (1.04-2.34) |  |
| Biombo | 184 (24) | 46 | reference | 0.0001 | reference | 0.0001 |
| Bijagós/Bolama | 49 (7) | 71 | 1.56 (1.23-1.98) |  | 2.65 (1.79-3.93) |  |
| Cacheu | 44 (6) | 41 | 0.90 (0.61-1.32) |  | 1.73 (1.07-2.80) |  |
| Gabu | 96 (13) | 79 | 1.73 (1.44-2.09) |  | 2.39 (1.59-3.59) |  |
| Oio | 57 (8) | 89 | 1.96 (1.64-2.35) |  | 2.15 (1.50-3.08) |  |
| Quinara | 91 (12) | 68 | 1.49 (1.21-1.84) |  | 2.54 (1.75-3.68) |  |
| Sao Domingos | 58 (8) | 62 | 1.36 (1.05-1.76) |  | 2.34 (1.54-3.56) |  |
| Tombali | 76 (10) | 53 | 1.15 (0.88-1.50) |  | 2.05 (1.34-3.15) |  |
| Village distance to vaccination facility (km) |  |  |  |  |  |  |
| ≥ 0, < 0.5 | 298 (39) | 63 | reference | 0.14 | reference | 0.11 |
| **Supplementary table S4 continued** | | | | | | |
| ≥ 0.5, < 2 | 114 (15) | 55 | 0.87 (0.72-1.05) |  | 0.95 (0.78-1.15) |  |
| ≥ 2, < 5 | 134 (18) | 53 | 0.84 (0.70-1.00) |  | 0.93 (0.78-1.11) |  |
| ≥ 5 | 210 (28) | 63 | 0.99 (0.87-1.13) |  | 1.14 (0.98-1.32) |  |
| Distance to sea (km) |  |  |  |  |  |  |
| ≥ 0, < 5 | 170 (23) | 55 | reference | 0.001 | - | - |
| ≥ 5, < 15 | 203 (27) | 53 | 0.95 (0.79-1.15) |  | - |  |
| ≥ 15, < 50 | 235 (31) | 62 | 1.12 (0.94-1.32) |  | - |  |
| ≥ 50, < 149 | 148 (20) | 74 | 1.33 (1.13-1.57) |  | - |  |
| Distance to hospital (km) |  |  |  |  |  |  |
| ≥ 0, < 10 | 147 (19) | 60 | reference | 0.03 | reference | 0.03 |
| ≥ 10, < 20 | 242 (32) | 67 | 1.13 (0.96-1.32) |  | 1.06 (0.88-1.27) |  |
| ≥ 20, < 30 | 217 (29) | 54 | 0.90 (0.75-1.08) |  | 0.85 (0.70-1.03) |  |
| ≥ 30, < 70 | 150 (20) | 58 | 0.97 (0.80-1.17) |  | 0.80 (0.63-1.02) |  |
| ***Use of health services*** |  |  |  |  |  |  |
| Most recent vaccination type^d,h^ |  |  |  |  |  |  |
| Inactivated | 454 (60) | 61 | reference | 0.62 | reference | 0.04 |
| Live | 95 (13) | 55 | 0.90 (0.74-1.10) |  | 0.79 (0.66-0.96) |  |
| Mixed | 111 (15) | 64 | 1.05 (0.90-1.23) |  | 1.08 (0.94-1.25) |  |
| No vaccination | 84 (11) | 61 | 1.00 (0.83-1.20) |  | 1.02 (0.84-1.22) |  |
| VAS received within 6 months^d^ |  |  |  |  |  |  |
| Received | 419 (55) | 59 | reference | 0.55 | - | - |
| Not received | 329 (44) | 61 | 0.96 (0.86-1.09) |  | - |  |
| Birth facility |  |  |  |  |  |  |
| Home | 420 (56) | 59 | reference | 0.01 | reference | 0.02 |
| Health unit/center | 55 (7) | 47 | 0.80 (0.60-1.07) |  | 0.97 (0.74-1.28) |  |
| Hospital | 91 (12) | 75 | 1.26 (1.09-1.45) |  | 1.24 (1.05-1.45) |  |
| No information | 190 (25) | 59 | 0.99 (0.86-1.15) |  | 1.16 (1.02-1.33) |  |
| Child use of bed net^d^ |  |  |  |  |  |  |
| Year around | 535 (71) | 62 | reference | 0.03 | reference | 0.06 |
| Rainy season | 206 (27) | 56 | 0.91 (0.79-1.04) |  | 0.98 (0.84-1.15) |  |
| None | 7 (1) | 86 | 1.39 (1.02-1.89) |  | 1.73 (1.10-2.73) |  |

^a^Poisson regression adjusted for CRP > 5 mg/L, ^b^Poisson regression adjusted for CRP > 5 mg/L, sex, age, weight for age, arm circumference for age, twinning status, cough on inclusion day, maternal major ethnic group, season, region, village distance to vaccination facility, distance to hospital, most recent vaccination type, birth facility, and child use of bed net. ^c^Wald test for overall equality of categories within variable. ^d^Numbers do not add up to 756 because records with missing information were discarded if they represent < 2 % of the 1102 inclusions, ^e^z-score in standard deviations from 1. ^f^p=0.87 when children missing information was omitted, ^g^Grouped from minor ethnicities. ^h^Vaccination status determined by the most recent vaccination type being either live (BCG, OPV, MV, and/or yellow fever vaccine), inactivated (DTP or Pentavalent vaccine) or Mixed (both a live and an inactivated vaccine). OPV is supposed to be given with DTP vaccines, but the last vaccine type was not considered as mixed if OPV was given with a DTP or Pentavalent vaccine as done in previous studies.

**Supplementary table S5.** Risk factors for vitamin A deficiency in rural Guinea-Bissau retaining only the most deficient children by setting the cut-off for VAD 10% lower than 0.83

| **Group/Variable** | **n (%)** | **VAD Prevalence (%)** | **Simple model^a^ PR for VAD (95%CI)** | **Simple model p^c^ <** | **Large model^b^ PR for VAD (95%CI)** | **Large model p^c^ <** |
| --- | --- | --- | --- | --- | --- | --- |
| VAD |  |  |  |  |  |  |
| Yes | 634 (42) | - | - | - | - | - |
| No | 468 (58) | - | - |  | - |  |
| ***Obligatory*** |  |  |  |  |  |  |
| Sex |  |  |  |  |  |  |
| Male | 555 (50) | 59 | reference | 0.53 | reference | 0.12 |
| Female | 547 (50) | 56 | 0.97 (0.88-1.07) |  | 0.93 (0.84-1.02) |  |
| Age |  |  |  |  |  |  |
| Continuous (months) | 1102 (100) | - | 0.98 (0.97-1.00) | 0.02 | 1.00 (0.98-1.01) | 0.51 |
| CRP |  |  |  |  |  |  |
| ≤ 5 mg/L | 756 (69) | 52 | reference | 0.0001 | reference | 0.0001 |
| > 5 mg/L | 346 (31) | 70 | 1.34 (1.22-1.48) |  | 1.31 (1.19-1.44) |  |
| ***Child factors*** |  |  |  |  |  |  |
| Weight for age^d,e^ |  |  |  |  |  |  |
| Continuous | 1094 (99) | - | 0.97 (0.93-1.02) | 0.22 | 1.04 (0.97-1.11) | 0.27 |
| Arm circumference for age^c,d^ |  |  |  |  |  |  |
| Continuous | 1101 (100) | - | 0.93 (0.89-0.98) | 0.01 | 0.94 (0.87-1.01) | 0.08 |
| Length for age^d,e^ |  |  |  |  |  |  |
| < -2 | 267 (24) | 61 | 0.99 (0.85-1.16) |  | - | - |
| ≥ -2, < -1 | 325 (30) | 52 | 0.87 (0.74-1.02) |  | - | - |
| ≥ -1, < 0 | 345 (31) | 59 | 0.95 (0.82-1.11) |  | - | - |
| ≥ 0 | 161 (15) | 61 | reference | 0.20 | - | - |
| Weight for length^c,e^ |  |  |  |  |  |  |
| Continuous | 1090 (99) | - | 0.98 (0.94-1.02) | 0.24 | - | - |
| Twinning status |  |  |  |  |  |  |
| Singletons | 1066 (97) | 57 | reference | 0.05 | reference | 0.23 |
| Twins | 36 (3) | 72 | 1.25 (1.05-1.55) |  | 1.17 (0.91-1.51) |  |
| Breast feeding^d^ |  |  |  |  |  |  |
| Yes | 1080 (98) | 58 | reference | 0.37 | - | - |
| Stopped | 17 (2) | 47 | 0.81 (0.50-1.29) |  | - |  |
| Took medicine on inclusion day |  |  |  |  |  |  |
| No | 975 (88) | 56 | reference | 0.01^f^ | - | - |
| Yes | 63 (6) | 65 | 1.08 (0.89-1.30) |  | - |  |
| No information | 64 (6) | 73 | 1.32 (1.13-1.54) |  | - |  |
| **Supplementary table S5 continued** | | | | | | |
| Cough at the day of inclusion^d^ |  |  |  |  |  |  |
| No | 816 (74) | 55 | reference | 0.01 | reference | 0.03 |
| Yes | 283 (26) | 65 | 1.15 (1.04-1.28) |  | 1.13 (1.01-1.26) |  |
| Fever at the day of inclusion^d^ |  |  |  |  |  |  |
| No | 758 (69) | 55 | reference | 0.09 | - | - |
| Yes | 341 (31) | 63 | 1.10 (0.99-1.22) |  | - |  |
| Diarrhea at the day of inclusion^d^ |  |  |  |  |  |  |
| No | 920 (83) | 57 | reference | 0.19 | - | - |
| Yes | 178 (16) | 63 | 1.09 (0.96-1.23) |  | - |  |
| ***Maternal factors*** |  |  |  |  |  |  |
| Maternal ethnicity^d^ |  |  |  |  |  |  |
| Balanta | 282 (26) | 54 | reference | 0.0001 | reference | 0.001 |
| Fula | 215 (20) | 75 | 1.41 (1.24-1.60) |  | 1.33 (1.09-1.61) |  |
| Mandinga | 123 (11) | 54 | 1.01 (0.83-1.23) |  | 0.89 (0.74-1.08) |  |
| Pepel | 235 (21) | 50 | 0.90 (0.77-1.06) |  | 1.51 (1.08-2.13) |  |
| Other^g^ | 234 (21) | 55 | 1.02 (0.87-1.19) |  | 0.99 (0.84-1.16) |  |
| Maternal educational level (years of school)^d^ |  |  |  |  |  |  |
| None | 797 (72) | 57 | reference | 0.72 | - | - |
| 1-4 | 197 (18) | 55 | 0.98 (0.86-1.13) |  | - |  |
| > 4 | 101 (9) | 61 | 1.06 (0.90-1.25) |  | - |  |
| Maternal age at child inclusion date^d^ |  |  |  |  |  |  |
| ≥ 14, ≤ 20 | 202 (18) | 58 | 0.99 (0.87-1.14) |  | - |  |
| > 20, ≤ 30 | 575 (52) | 58 | reference | 0.99 | - | - |
| > 30, ≤ 40 | 260 (24) | 58 | 1.01 (0.89-1.14) |  | - |  |
| > 40, ≤ 55 | 43 (4) | 53 | 0.96 (0.72-1.27) |  | - |  |
| ***Season*** |  |  |  |  |  |  |
| Season |  |  |  |  |  |  |
| Dry | 649 (59) | 43 | reference | 0.0001 | reference | 0.0001 |
| Rainy | 453 (49) | 78 | 1.80 (1.63-1.98) |  | 1.98 (1.77-2.22) |  |
| ***Geography*** |  |  |  |  |  |  |
| Region |  |  |  |  |  |  |
| Bafata | 121 (11) | 49 | 1.14 (0.91-1.42) |  | 1.57 (1.07-2.32) |  |
| Biombo | 287 (26) | 45 | reference | 0.0001 | reference | 0.0001 |
| Bijagós/Bolama | 63 (6) | 67 | 1.53 (1.24-1.89) |  | 3.18 (2.10-4.81) |  |
| Cacheu | 53 (5) | 36 | 0.84 (0.57-1.22) |  | 1.72 (1.05-2.80) |  |
| Gabu | 148 (13) | 74 | 1.64 (1.41-1.92) |  | 2.49 (1.68-3.68) |  |
| Oio | 89 (8) | 82 | 1.81 (1.54-2.12) |  | 2.18 (1.52-3.11) |  |
| Quinara | 133 (12) | 62 | 1.38 (1.15-1.66) |  | 2.36 (1.64-3.40) |  |
| Sao Domingos | 91 (8) | 59 | 1.31 (1.06-1.61) |  | 2.20 (1.51-3.22) |  |
| Tombali | 117 (11) | 56 | 1.23 (1.01-1.50) |  | 2.43 (1.64-3.60) |  |
| Village distance to vaccination facility (km) |  |  |  |  |  |  |
| ≥ 0, < 0.5 | 440 (40) | 62 | reference | 0.02 | reference | 0.18 |
| ≥ 0.5, < 2 | 167 (15) | 52 | 0.85 (0.72-1.00) |  | 0.95 (0.80-1.12) |  |
| **Supplementary table S5 continued** | | | | | | |
| ≥ 2, < 5 | 200 (18) | 50 | 0.81 (0.69-0.95) |  | 0.92 (0.80-1.07) |  |
| ≥ 5 | 295 (27) | 60 | 0.98 (0.87-1.10) |  | 1.09 (0.96-1.24) |  |
|  | |  |  |  |  |  |
| Distance to sea (km) |  |  |  |  |  |  |
| ≥ 0, < 5 | 25 9 (24) | 52 | reference | 0.0001 | - | - |
| ≥ 5, < 15 | 290 (26) | 52 | 0.98 (0.84-1.15) |  | - |  |
| ≥ 15, < 50 | 328 (30) | 58 | 1.11 (0.96-1.28) |  | - |  |
| ≥ 50, < 149 | 225 (20) | 70 | 1.31 (1.15-1.51) |  | - |  |
| Distance to hospital (km) |  |  |  |  |  |  |
| ≥ 0, < 10 | 206 (19) | 54 | reference | 0.19 | reference | 0.10 |
| ≥ 10, < 20 | 363 (33) | 62 | 1.14 (0.98-1.32) |  | 1.12 (0.95-1.31) |  |
| ≥ 20, < 30 | 319 (29) | 55 | 1.01 (0.86-1.19) |  | 1.02 (0.86-1.21) |  |
| ≥ 30, < 70 | 214 (19) | 57 | 1.05 (0.88-1.24) |  | 0.89 (0.73-1.10) |  |
| ***Use of health services*** |  |  |  |  |  |  |
| Most recent vaccination type^d,h^ |  |  |  |  |  |  |
| Inactivated | 667 (61) | 58 | reference | 0.50 | reference | 0.01 |
| Live | 141 (13) | 53 | 0.91 (0.77-1.07) |  | 0.77 (0.65-0.91) |  |
| Mixed | 156 (14) | 61 | 1.05 (0.91-1.21) |  | 1.08 (0.95-1.23) |  |
| No vaccination | 119 (11) | 55 | 0.96 (0.81-1.13) |  | 0.96 (0.81-1.14) |  |
| VAS received within 6 months^d^ |  |  |  |  |  |  |
| Received | 589 (53) | 55 | reference | 0.14 | - | - |
| Not received | 500 (45) | 60 | 1.08 (0.98-1.20) |  | - |  |
| Birth facility |  |  |  |  |  |  |
| Home | 629 (57) | 58 | reference | 0.09 | reference | 0.14 |
| Health unit/center | 80 (7) | 49 | 0.85 (0.68-1.06) |  | 1.01 (0.83-1.22) |  |
| Hospital | 126 (11) | 65 | 1.14 (0.99-1.32) |  | 1.08 (0.93-1.27) |  |
| No information | 267 (24) | 56 | 0.98 (0.86-1.11) |  | 1.15 (1.02-1.29) |  |
| Child use of bed net^d^ |  |  |  |  |  |  |
| Year around | 773 (70) | 59 | reference | 0.001 | reference | 0.01 |
| Rainy season | 311 (28) | 54 | 0.90 (0.80-1.01) |  | 0.93 (0.81-1.07) |  |
| None | 8 (1) | 88 | 1.57 (1.20-2.05) |  | 1.77 (1.26-2.48) |  |

^a^Poisson regression adjusted for CRP > 5 mg/L, ^b^Poisson regression adjusted for CRP > 5 mg/L, sex, age, weight for age, arm circumference for age, twinning status, cough on inclusion day, maternal major ethnic group, season, region, village distance to vaccination facility, distance to hospital, most recent vaccination type, birth facility, and child use of bed net. ^c^Wald test for overall equality of categories within variable. ^d^Numbers do not add up to 1102 because records with missing information were discarded if they represent < 2 %, ^e^z-score in standard deviations from 1. ^f^p=0.46 when children missing information was omitted, ^g^Grouped from minor ethnicities: ^g^Grouped from minor ethnicities. ^h^Vaccination status determined by the most recent vaccination type being either live (BCG, OPV, MV, and/or yellow fever vaccine), inactivated (DTP or Pentavalent vaccine) or Mixed (both a live and an inactivated vaccine). OPV is supposed to be given with DTP vaccines, but the last vaccine type was not considered as mixed if OPV was given with a DTP or Pentavalent vaccine as done in previous studies.

**Supplementary table S6.** Risk factors for vitamin A deficiency in rural Guinea-Bissau retaining both the most and marginally deficient children by setting the cut-off for VAD 10% higher than 0.83

| **Group/Variable** | **n (%)** | **VAD Prevalence (%)** | **Simple model^a^ PR for VAD (95%CI)** | **Simple model p^c^ <** | **Large model^b^ PR for VAD (95%CI)** | **Large model p^c^ <** |
| --- | --- | --- | --- | --- | --- | --- |
| VAD |  |  |  |  |  |  |
| Yes | 810 (74) | - | - | - | - | - |
| No | 292 (27) | - | - |  | - |  |
| ***Obligatory*** |  |  |  |  |  |  |
| Sex |  |  |  |  |  |  |
| Male | 555 (50) | 73 | reference | 0.95 | reference | 0.25 |
| Female | 547 (50) | 74 | 1.00 (0.93-1.07) |  | 0.96 (0.90-1.03) |  |
| Age |  |  |  |  |  |  |
| Continuous (months) | 1102 (100) | - | 0.99 (0.99-1.00) | 0.02 | 1.00 (0.99-1.01) | 0.72 |
| CRP |  |  |  |  |  |  |
| ≤ 5 mg/L | 756 (69) | 69 | reference | 0.0001 | reference | 0.0001 |
| > 5 mg/L | 346 (31) | 84 | 1.23 (1.15-1.31) |  | 1.20 (1.12-1.28) |  |
| ***Child factors*** |  |  |  |  |  |  |
| Weight for age^d,e^ |  |  |  |  |  |  |
| Continuous | 1094 (99) | - | 1.01 (0.98-1.04) | 0.62 | 1.04 (0.98-1.09) | 0.18 |
| Arm circumference for age^c,d^ |  |  |  |  |  |  |
| Continuous | 1101 (100) | - | 0.99 (0.96-1.02) | 0.49 | 0.99 (0.94-1.04) | 0.68 |
| Length for age^d,e^ |  |  |  |  |  |  |
| < -2 | 267 (24) | 75 | 1.00 (0.90-1.12) |  | - | - |
| ≥ -2, < -1 | 325 (30) | 70 | 0.94 (0.84-1.05) |  | - | - |
| ≥ -1, < 0 | 345 (31) | 76 | 1.01 (0.91-1.13) |  | - | - |
| ≥ 0 | 161 (15) | 75 | reference | 0.42 | - | - |
| Weight for length^c,e^ |  |  |  |  |  |  |
| Continuous | 1090 (99) | - | 1.01 (0.98-1.04) | 0.64 | - | - |
| Twinning status |  |  |  |  |  |  |
| Singletons | 1066 (97) | 73 | reference | 0.01 | reference | 0.01 |
| Twins | 36 (3) | 89 | 1.21 (1.07-1.36) |  | 1.22 (1.06-1.40) |  |
| Breast feeding^d^ |  |  |  |  |  |  |
| Yes | 1080 (98) | 74 | reference | 0.81 | - | - |
| Stopped | 17 (2) | 76 | 1.03 (0.80-1.33) |  | - |  |
| Took medicine on inclusion day |  |  |  |  |  |  |
| No | 975 (88) | 73 | reference | 0.08^f^ | - | - |
| Yes | 63 (6) | 75 | 0.97 (0.84-1.13) |  | - |  |
| No information | 64 (6) | 83 | 1.14 (1.01-1.29) |  | - |  |
| **Supplementary table S6 continued** | | | | | | |
| Cough at the day of inclusion^d^ |  |  |  |  |  |  |
| No | 816 (74) | 71 | reference | 0.01 | reference | 0.01 |
| Yes | 283 (26) | 80 | 1.11 (1.03-1.19) |  | 1.11 (1.04-1.19) |  |
| Fever at the day of inclusion^d^ |  |  |  |  |  |  |
| No | 758 (69) | 72 | reference | 0.56 | - | - |
| Yes | 341 (31) | 76 | 1.02 (0.95-1.10) |  | - |  |
| Diarrhea at the day of inclusion^d^ |  |  |  |  |  |  |
| No | 920 (83) | 73 | reference | 0.39 | - | - |
| Yes | 178 (16) | 77 | 1.04 (0.95-1.14) |  | - |  |
| ***Maternal factors*** |  |  |  |  |  |  |
| Maternal ethnicity^d^ |  |  |  |  |  |  |
| Balanta | 282 (26) | 72 | reference | 0.0001 | reference | 0.01 |
| Fula | 215 (20) | 84 | 1.17 (1.07-1.29) |  | 1.07 (0.94-1.22) |  |
| Mandinga | 123 (11) | 67 | 0.94 (0.81-1.08) |  | 0.87 (0.76-0.99) |  |
| Pepel | 235 (21) | 71 | 0.97 (0.87-1.08) |  | 1.28 (1.02-1.59) |  |
| Other^g^ | 234 (21) | 71 | 0.98 (0.88-1.10) |  | 0.94 (0.84-1.06) |  |
| Maternal educational level (years of school)^d^ |  |  |  |  |  |  |
| None | 797 (72) | 74 | reference | 0.62 | - | - |
| 1-4 | 197 (18) | 70 | 0.96 (0.87-1.06) |  | - |  |
| > 4 | 101 (9) | 76 | 1.03 (0.92-1.15) |  | - |  |
| Maternal age at child inclusion date^d^ |  |  |  |  |  |  |
| ≥ 14, ≤ 20 | 202 (18) | 74 | 1.00 (0.91-1.10) |  | - |  |
| > 20, ≤ 30 | 575 (52) | 73 | reference | 1.00 | - | - |
| > 30, ≤ 40 | 260 (24) | 74 | 1.01 (0.92-1.10) |  | - |  |
| > 40, ≤ 55 | 43 (4) | 72 | 1.01 (0.83-1.22) |  | - |  |
| ***Season*** |  |  |  |  |  |  |
| Season |  |  |  |  |  |  |
| Dry | 649 (59) | 62 | reference | 0.0001 | reference | 0.0001 |
| Rainy | 453 (49) | 90 | 1.44 (1.35-1.54) |  | 1.51 (1.39-1.63) |  |
| ***Geography*** |  |  |  |  |  |  |
| Region |  |  |  |  |  |  |
| Bafata | 121 (11) | 62 | 0.97 (0.83-1.14) |  | 1.31 (1.01-1.71) |  |
| Biombo | 287 (26) | 66 | reference | 0.0001 | reference | 0.0001 |
| Bijagós/Bolama | 63 (6) | 87 | 1.35 (1.20-1.53) |  | 2.07 (1.60-2.68) |  |
| Cacheu | 53 (5) | 49 | 0.77 (0.58-1.02) |  | 1.25 (0.89-1.77) |  |
| Gabu | 148 (13) | 86 | 1.30 (1.17-1.44) |  | 1.84 (1.42-2.39) |  |
| Oio | 89 (8) | 97 | 1.46 (1.33-1.60) |  | 1.78 (1.42-2.22) |  |
| Quinara | 133 (12) | 74 | 1.13 (1.00-1.29) |  | 1.66 (1.30-2.11) |  |
| Sao Domingos | 91 (8) | 69 | 1.05 (0.89-1.23) |  | 1.58 (1.23-2.04) |  |
| Tombali | 117 (11) | 76 | 1.15 (1.01-1.31) |  | 1.89 (1.46-2.44) |  |
| Village distance to vaccination facility (km) |  |  |  |  |  |  |
| ≥ 0, < 0.5 | 440 (40) | 75 | reference | 0.48 | reference | 0.65 |
| ≥ 0.5, < 2 | 167 (15) | 72 | 0.95 (0.86-1.06) |  | 0.97 (0.86-1.09) |  |
| **Supplementary table S6 continued** | | | | | | |
| ≥ 2, < 5 | 200 (18) | 70 | 0.93 (0.84-1.03) |  | 0.99 (0.89-1.09) |  |
| ≥ 5 | 295 (27) | 74 | 0.99 (0.91-1.07) |  | 1.04 (0.95-1.14) |  |
| Distance to sea (km) |  |  |  |  |  |  |
| ≥ 0, < 5 | 25 9 (24) | 72 | reference | 0.20 | - | - |
| ≥ 5, < 15 | 290 (26) | 72 | 1.01 (0.91-1.12) |  | - |  |
| ≥ 15, < 50 | 328 (30) | 72 | 1.01 (0.91-1.12) |  | - |  |
| ≥ 50, < 149 | 225 (20) | 79 | 1.10 (0.99-1.21) |  | - |  |
| Distance to hospital (km) |  |  |  |  |  |  |
| ≥ 0, < 10 | 206 (19) | 74 | reference | 0.03 | reference | 0.05 |
| ≥ 10, < 20 | 363 (33) | 79 | 1.05 (0.96-1.15) |  | 1.02 (0.91-1.14) |  |
| ≥ 20, < 30 | 319 (29) | 69 | 0.93 (0.83-1.03) |  | 0.91 (0.81-1.03) |  |
| ≥ 30, < 70 | 214 (19) | 70 | 0.94 (0.84-1.06) |  | 0.88 (0.76-1.01) |  |
| ***Use of health services*** |  |  |  |  |  |  |
| Most recent vaccination type^d,h^ |  |  |  |  |  |  |
| Inactivated | 667 (61) | 74 | reference | 0.60 | reference | 0.02 |
| Live | 141 (13) | 72 | 0.98 (0.88-1.09) |  | 0.88 (0.79-0.97) |  |
| Mixed | 156 (14) | 72 | 0.99 (0.89-1.10) |  | 0.98 (0.89-1.09) |  |
| No vaccination | 119 (11) | 78 | 1.07 (0.96-1.18) |  | 1.09 (0.99-1.21) |  |
| VAS received within 6 months^d^ |  |  |  |  |  |  |
| Received | 589 (53) | 71 | reference | 0.19 | - | - |
| Not received | 500 (45) | 76 | 1.05 (0.98-1.03) |  | - |  |
| Birth facility |  |  |  |  |  |  |
| Home | 629 (57) | 74 | reference | 0.01 | reference | 0.10 |
| Health unit/center | 80 (7) | 65 | 0.89 (0.75-1.04) |  | 0.95 (0.82-1.10) |  |
| Hospital | 126 (11) | 83 | 1.13 (1.03-1.25) |  | 1.10 (1.00-1.22) |  |
| No information | 267 (24) | 72 | 0.98 (0.90-1.07) |  | 1.07 (0.98-1.17) |  |
| Child use of bed net^d^ |  |  |  |  |  |  |
| Year around | 773 (70) | 74 | reference | 0.21 | reference | 0.18 |
| Rainy season | 311 (28) | 72 | 0.97 (0.90-1.05) |  | 1.02 (0.93-1.12) |  |
| None | 8 (1) | 88 | 1.23 (0.94-1.60) |  | 1.26 (0.98-1.62) |  |

^a^Poisson regression adjusted for CRP > 5 mg/L, ^b^Poisson regression adjusted for CRP > 5 mg/L, sex, age, weight for age, arm circumference for age, twinning status, cough on inclusion day, maternal major ethnic group, season, region, village distance to vaccination facility, distance to hospital, most recent vaccination type, birth facility, and child use of bed net. ^c^Wald test for overall equality of categories within variable. ^d^Numbers do not add up to 1102 because records with missing information were discarded if they represent < 2 %, ^e^z-score in standard deviations from 1. ^f^p=0.68 when children missing information was omitted, ^g^Grouped from minor ethnicities: ^g^Grouped from minor ethnicities. ^h^Vaccination status determined by the most recent vaccination type being either live (BCG, OPV, MV, and/or yellow fever vaccine), inactivated (DTP or Pentavalent vaccine) or Mixed (both a live and an inactivated vaccine). OPV is supposed to be given with DTP vaccines, but the last vaccine type was not considered as mixed if OPV was given with a DTP or Pentavalent vaccine as done in previous studies.
